# Supplementary material for: Pyruvate Dehydrogenase Kinase 1 inhibition mediated oxidative phosphorylation enhancement in cartilage promotes osteoarthritis progression
Source: BMC Musculoskelet Disord. 2023 Jul 20;24:597. doi: 10.1186/s12891-023-06585-6 (PMC10357736; doi:10.1186/s12891-023-06585-6)
Supplement: Supplementary file 1 — Additional file 1. [file 12891_2023_6585_MOESM1_ESM.zip › KEGG.Gsea.1653622432464/KEGG_HEDGEHOG_SIGNALING_PATHWAY.html]

Details for gene set KEGG\_HEDGEHOG\_SIGNALING\_PATHWAY[GSEA]

|  || Dataset | 55457\_collapsed\_to\_symbols.cls.cls #OA\_versus\_normal.cls.cls #OA\_versus\_normal\_repos |
| Phenotype | cls.cls#OA\_versus\_normal\_repos |
| Upregulated in class | normal |
| GeneSet | KEGG\_HEDGEHOG\_SIGNALING\_PATHWAY |
| Enrichment Score (ES) | -0.3639035 |
| Normalized Enrichment Score (NES) | -1.3691044 |
| Nominal p-value | 0.06724138 |
| FDR q-value | 0.25447413 |
| FWER p-Value | 0.998 |
Table: GSEA Results Summary

  

Fig 1: Enrichment plot: KEGG\_HEDGEHOG\_SIGNALING\_PATHWAY      
 Profile of the Running ES Score & Positions of GeneSet Members on the Rank Ordered List

  

| SYMBOL | TITLE | RANK IN GENE LIST | RANK METRIC SCORE | RUNNING ES | CORE ENRICHMENT || 1 | WNT5B | Wnt family member 5B [Source:HGNC Symbol;Acc:HGNC:16265] | 327 | 0.734 | 0.0215 | No |
| 2 | CSNK1G3 | casein kinase 1 gamma 3 [Source:HGNC Symbol;Acc:HGNC:2456] | 705 | 0.592 | 0.0300 | No |
| 3 | PRKACB | protein kinase cAMP-activated catalytic subunit beta [Source:HGNC Symbol;Acc:HGNC:9381] | 771 | 0.576 | 0.0618 | No |
| 4 | RAB23 | "RAB23, member RAS oncogene family [Source:HGNC Symbol;Acc:HGNC:14263]" | 1225 | 0.471 | 0.0567 | No |
| 5 | WNT5A | Wnt family member 5A [Source:HGNC Symbol;Acc:HGNC:12784] | 1995 | 0.361 | 0.0200 | No |
| 6 | WNT3 | Wnt family member 3 [Source:HGNC Symbol;Acc:HGNC:12782] | 2003 | 0.360 | 0.0425 | No |
| 7 | WNT2 | Wnt family member 2 [Source:HGNC Symbol;Acc:HGNC:12780] | 2700 | 0.279 | 0.0062 | No |
| 8 | GLI3 | GLI family zinc finger 3 [Source:HGNC Symbol;Acc:HGNC:4319] | 2861 | 0.264 | 0.0107 | No |
| 9 | LRP2 | LDL receptor related protein 2 [Source:HGNC Symbol;Acc:HGNC:6694] | 3236 | 0.227 | -0.0039 | No |
| 10 | GAS1 | growth arrest specific 1 [Source:HGNC Symbol;Acc:HGNC:4165] | 3757 | 0.181 | -0.0328 | No |
| 11 | WNT2B | Wnt family member 2B [Source:HGNC Symbol;Acc:HGNC:12781] | 4194 | 0.138 | -0.0579 | No |
| 12 | CSNK1D | casein kinase 1 delta [Source:HGNC Symbol;Acc:HGNC:2452] | 4529 | 0.111 | -0.0768 | No |
| 13 | WNT11 | Wnt family member 11 [Source:HGNC Symbol;Acc:HGNC:12776] | 4688 | 0.097 | -0.0829 | No |
| 14 | WNT6 | Wnt family member 6 [Source:HGNC Symbol;Acc:HGNC:12785] | 5781 | 0.010 | -0.1672 | No |
| 15 | WNT7B | Wnt family member 7B [Source:HGNC Symbol;Acc:HGNC:12787] | 6230 | -0.023 | -0.2006 | No |
| 16 | BMP8A | bone morphogenetic protein 8a [Source:HGNC Symbol;Acc:HGNC:21650] | 6537 | -0.045 | -0.2215 | No |
| 17 | WNT7A | Wnt family member 7A [Source:HGNC Symbol;Acc:HGNC:12786] | 7166 | -0.093 | -0.2644 | No |
| 18 | WNT10B | Wnt family member 10B [Source:HGNC Symbol;Acc:HGNC:12775] | 7700 | -0.134 | -0.2973 | No |
| 19 | BMP4 | bone morphogenetic protein 4 [Source:HGNC Symbol;Acc:HGNC:1071] | 7816 | -0.142 | -0.2971 | No |
| 20 | BMP5 | bone morphogenetic protein 5 [Source:HGNC Symbol;Acc:HGNC:1072] | 8488 | -0.193 | -0.3370 | No |
| 21 | WNT16 | Wnt family member 16 [Source:HGNC Symbol;Acc:HGNC:16267] | 8808 | -0.218 | -0.3479 | Yes |
| 22 | PTCH1 | patched 1 [Source:HGNC Symbol;Acc:HGNC:9585] | 8837 | -0.220 | -0.3360 | Yes |
| 23 | SHH | sonic hedgehog signaling molecule [Source:HGNC Symbol;Acc:HGNC:10848] | 8941 | -0.227 | -0.3295 | Yes |
| 24 | PTCH2 | patched 2 [Source:HGNC Symbol;Acc:HGNC:9586] | 8994 | -0.231 | -0.3188 | Yes |
| 25 | CSNK1G1 | casein kinase 1 gamma 1 [Source:HGNC Symbol;Acc:HGNC:2454] | 9378 | -0.262 | -0.3318 | Yes |
| 26 | BMP7 | bone morphogenetic protein 7 [Source:HGNC Symbol;Acc:HGNC:1074] | 9544 | -0.278 | -0.3269 | Yes |
| 27 | BMP6 | bone morphogenetic protein 6 [Source:HGNC Symbol;Acc:HGNC:1073] | 10021 | -0.323 | -0.3432 | Yes |
| 28 | SMO | "smoothened, frizzled class receptor [Source:HGNC Symbol;Acc:HGNC:11119]" | 10111 | -0.332 | -0.3289 | Yes |
| 29 | BMP8B | bone morphogenetic protein 8b [Source:HGNC Symbol;Acc:HGNC:1075] | 10419 | -0.363 | -0.3296 | Yes |
| 30 | PRKACG | protein kinase cAMP-activated catalytic subunit gamma [Source:HGNC Symbol;Acc:HGNC:9382] | 10466 | -0.370 | -0.3095 | Yes |
| 31 | PRKACA | protein kinase cAMP-activated catalytic subunit alpha [Source:HGNC Symbol;Acc:HGNC:9380] | 10611 | -0.384 | -0.2962 | Yes |
| 32 | WNT1 | Wnt family member 1 [Source:HGNC Symbol;Acc:HGNC:12774] | 10706 | -0.394 | -0.2783 | Yes |
| 33 | BTRC | beta-transducin repeat containing E3 ubiquitin protein ligase [Source:HGNC Symbol;Acc:HGNC:1144] | 10836 | -0.409 | -0.2622 | Yes |
| 34 | IHH | Indian hedgehog signaling molecule [Source:HGNC Symbol;Acc:HGNC:5956] | 10886 | -0.415 | -0.2394 | Yes |
| 35 | BMP2 | bone morphogenetic protein 2 [Source:HGNC Symbol;Acc:HGNC:1069] | 10919 | -0.420 | -0.2151 | Yes |
| 36 | GSK3B | glycogen synthase kinase 3 beta [Source:HGNC Symbol;Acc:HGNC:4617] | 11157 | -0.451 | -0.2046 | Yes |
| 37 | GLI2 | GLI family zinc finger 2 [Source:HGNC Symbol;Acc:HGNC:4318] | 11195 | -0.456 | -0.1783 | Yes |
| 38 | CSNK1A1 | casein kinase 1 alpha 1 [Source:HGNC Symbol;Acc:HGNC:2451] | 11310 | -0.472 | -0.1570 | Yes |
| 39 | CSNK1E | casein kinase 1 epsilon [Source:HGNC Symbol;Acc:HGNC:2453] | 11491 | -0.500 | -0.1390 | Yes |
| 40 | WNT8B | Wnt family member 8B [Source:HGNC Symbol;Acc:HGNC:12789] | 11532 | -0.505 | -0.1098 | Yes |
| 41 | PRKX | protein kinase X-linked [Source:HGNC Symbol;Acc:HGNC:9441] | 11634 | -0.522 | -0.0843 | Yes |
| 42 | FBXW11 | F-box and WD repeat domain containing 11 [Source:HGNC Symbol;Acc:HGNC:13607] | 11982 | -0.590 | -0.0735 | Yes |
| 43 | WNT4 | Wnt family member 4 [Source:HGNC Symbol;Acc:HGNC:12783] | 12331 | -0.686 | -0.0567 | Yes |
| 44 | GLI1 | GLI family zinc finger 1 [Source:HGNC Symbol;Acc:HGNC:4317] | 12333 | -0.687 | -0.0129 | Yes |
| 45 | CSNK1G2 | casein kinase 1 gamma 2 [Source:HGNC Symbol;Acc:HGNC:2455] | 12729 | -0.890 | 0.0133 | Yes |
Table: GSEA details [plain text format]

  

Fig 2: KEGG\_HEDGEHOG\_SIGNALING\_PATHWAY      
 Blue-Pink O' Gram in the Space of the Analyzed GeneSet

  

Fig 3: KEGG\_HEDGEHOG\_SIGNALING\_PATHWAY: Random ES distribution      
 Gene set null distribution of ES for **KEGG\_HEDGEHOG\_SIGNALING\_PATHWAY**
